# Individual differences in rhythm perception modulate music-related motor learning: a neurobehavioral training study with children

**DOI:** 10.1038/s41598-023-48132-2

**Published:** 2023-12-06

**Authors:** Marta Martins, Ana Mafalda Reis, Christian Gaser, São Luís Castro

**Affiliations:** 1 University Institute of Lisbon (ISCTE-IUL), 1649-026 Lisboa, Portugal; 2 Center for Psychology, Faculty of Psychology and Education Sciences, University of Porto, 4200-319 Porto, Portugal; 3 Unilabs Boavista, 4250-201 Porto, Portugal; 4 Department of Psychiatry and Psychotherapy, Jena University Hospital, 07743 Jena, Germany; 5 Department of Neurology, Jena University Hospital, 07743 Jena, Germany

**Keywords:** Human behaviour, Learning and memory

## Abstract

Rhythm and motor function are intrinsically linked to each other and to music, but the rhythm-motor interplay during music training, and the corresponding brain mechanisms, are underexplored. In a longitudinal training study with children, we examined the role of rhythm predisposition in the fine motor improvements arising from music training, and which brain regions would be implicated. Fifty-seven 8-year-olds were assigned to either a 6-month music training (n = 21), sports training (n = 18), or a control group (n = 18). They performed rhythm and motor tasks, and structural brain scans before and after training were collected. Better ability to perceive rhythm before training was related to less gray matter volume in regions of the cerebellum, fusiform gyrus, supramarginal gyrus, ventral diencephalon, amygdala, and inferior/middle temporal gyri. Music training improved motor performance, and greater improvements correlated with better pre-training rhythm discrimination. Music training also induced a loss of gray matter volume in the left cerebellum and fusiform gyrus, and volume loss correlated with higher motor gains. No such effects were found in the sports and control groups. In summary, children with finer-tuned rhythm perception abilities were prone to finer motor improvements through music training, and this rhythm-motor link was to some extent subserved by the left cerebellum and fusiform gyrus. These findings have implications for models on music-related plasticity and rhythm cognition, and for programs targeting motor function.

## Introduction

Rhythm is a core component of human biology. It underlies a wide variety of complex behaviors, such as speech, music, motor control, and social interaction, and is decisive for typical development^1^. Rhythm is especially linked with musical behaviors, such as synchronizing movement with musical rhythmic patterns (a series of time intervals created by sounds and silences that are regularly patterned in time^2^). This regularity favors the prediction of upcoming sonic events and the effortless synchronization with the music, or with whom one is playing or dancing^3,4^. *Perceiving rhythm* in music involves extracting a beat from the sound pattern, which then allows the perceiver to organize the upcoming events and sense their underlying metrical structure^2,5,6^. By comparison, *playing a musical instrument* or dancing involve components of rhythm cognition beyond perception, including synchronizing an internal cognitive pulse to the pulse of the external sound patterns, and using this synchronization to drive the motor output^7^. The triadic link between rhythm, music, and motor coordination, is the focus of this study. We ask if rhythm abilities play a role in the fine motor improvements that result from music training, and which brain areas would subtend such a putative triadic link.

The neural circuit implicated in cognizing rhythm in music is well described^2,8^. It consists of a widely distributed cortical and subcortical network that subserves sensory, motor, and cognitive aspects of rhythm processing and includes the supplementary motor area, the premotor, auditory, and inferior parietal cortices, and the basal ganglia and cerebellum. These regions have been implicated in slightly different aspects of rhythm cognition. For instance, the basal ganglia are claimed to be at the core of beat-based timing^9,10^; the premotor cortex contributes to sensorimotor integration^11,12^, and the cerebellum subserves complex processes, such as determining the duration of discrete stimuli^10,13^, correcting timing errors^10,14^, and synchronizing perception and action^15,16^. Interestingly, playing music without auditory feedback activates the auditory cortex^17^ and, conversely, motor regions, namely the premotor cortex and cerebellum, are implicated in rhythm processing even when the task only requires listening to auditory rhythms^9,18–22^. These findings suggest that auditory and motor processes are functionally coupled; it is this coupling that explains, at least in part, our compulsion to move when listening to a musical rhythm^6^.

Playing a musical instrument requires the precise perception and production of rhythmic patterns and their integration with motor commands. Music training is, therefore, a valuable framework for studying rhythm cognition, especially the interplay between auditory perception and motor actions^12,23^. Music training is associated with enhanced rhythm skills, including rhythm discrimination^24–27^ and production^24,28,29^. It is also associated with enhanced motor skills, such as the movements of the fingers, hands, and arms^30–34^. Several longitudinal studies have corroborated the causal nature of this relationship for rhythm^35–37^, as well as for motor skills^36,38–40^. Concordant with behavioral findings, cross-sectional neuroimaging studies have shown that musicians differ from non-musicians in the brain organization of the auditory-motor system^31,41–49; for reviews,23,50^. Furthermore, a growing body of longitudinal research suggests that music training drives auditory-motor plasticity at structural^36,51–53^ and functional levels^52,54–68^.

Because auditory rhythm and motor functions are closely intertwined in music performance^19,28; for a review, 12^, it is reasonable to conceive that music training modulates these skills not only one by one (independently) but also in their interaction. Indeed, musicians show a stronger auditory-motor coupling than non-musicians^17,69,70^. More generally, rhythm-based auditory input guides motor actions^71–73^, and the link between rhythm auditory cueing and movement has been valuable in the rehabilitation of movement^74,75^ and language disorders^76,77^ through music. However, in spite of this accumulated knowledge, evidence on how the predisposition to perceive auditory rhythm might drive motor learning associated with music-related practice is limited to rehabilitation studies with adult clinical populations^75,78^. Interestingly, these studies have uncovered that patients’ response to training, that is, its success or unsuccessfulness, depends on auditory predispositions and prior experience. For instance, in studies with Parkinson’s disease patients, Dalla Bella et al.^75^ have shown that walking to music improved gait and rhythm skills, but the response to training depended on the patients’ sensorimotor rhythmic skills prior to training; similarly, Cochen De Cock et al.^78^ found that the patients who responded positively to rhythm auditory cueing were more musically trained and had better rhythm and music perception skills than patients who did not have a positive response. These findings underline the role of individual differences in motor learning and add to the neurophysiological evidence showing that preexisting differences in brain structure and function can influence how fast and successful motor learning is^79–81^.

As indicated before, here we investigate the interplay between rhythm and motor skills in the context of music training, at behavioral and brain levels. In a longitudinal training study with children, we inspect whether the ability to perceive or reproduce rhythm (predisposition) modulates the motor improvements associated with music training, and determine brain regions implicated in the putative links between rhythm and motor learning. To this end, we include measures of rhythm perception and production, fine motor dexterity and coordination, and gray matter volume, and analyze them as follows. First, we inspect gray matter correlates of rhythm abilities. Then, we examine the effects of music training on motor skills, and whether putative benefits of music training on motor skills are related to pre-training rhythm abilities. After, we inspect the effects of music training on rhythm abilities and the relationship between rhythm and motor performance before training. Finally, we test whether the gray matter correlates of rhythm abilities are modulated by training and, if yes, whether they covary with the extent of motor improvement. Considering the established link between auditory rhythm and motor function^2,6^, and the involvement of motor regions in rhythm perception^9,19,22^, we hypothesize that the gray matter correlates of rhythm abilities include regions from both auditory and motor systems, and that rhythm abilities may facilitate motor learning. Extrapolating from findings in rehabilitation studies^75,78^, we expect that music training enhances motor learning especially in children with better rhythm predisposition.

## Results

### Gray matter correlates of rhythm skills before training

At pre-test, better rhythm discrimination was related to less gray matter volume in six clusters (Table 1): left cerebellum and fusiform gyrus (*x* =  − 24, *y* =  − 57, *z* =  − 18), left/right ventral diencephalon (*x* = 2, *y* =  − 2, *z* =  − 6), left supramarginal gyrus (*x* =  − 51, *y* =  − 44, *z* = 48), right cerebellum (*x* = 32, *y* =  − 81, *z* =  − 33), right ventral diencephalon and amygdala (*x* = 15, *y* =  − 4, *z* =  − 14), and left inferior/middle temporal gyri (*x* =  − 56, *y* =  − 68, *z* =  − 6). The whole brain analysis showed no significant positive correlations between gray matter volume and rhythm discrimination. No gray matter correlates were found for rhythm copy.

**Table 1:** Gray matter correlates of rhythm discrimination at pre-test (FDR-corrected, *p* < *.05, k* > *20*).

Cluster	Region		MNI coordinates of peak voxel			Cluster size (*k*)	*p*	TFCE
			*x*	*y*	*z*			
1	Cerebellum/FuG	L	− 24	− 57	− 18	6652	.017	147,728.80
2	Ventral DC	L/R	2	− 2	− 6	463	.017	58,012.98
3	SMG	L	− 51	− 44	48	516	.017	38,730.93
4	Cerebellum	R	32	− 81	− 33	722	.035	38,008.99
5	Ventral DC/Amygdala	R	15	− 4	− 14	145	.035	17,485.30
6	ITG/ MTG	L	− 56	− 68	− 6	167	.047	15,488.00

*FuG* Fusiform Gyrus, *DC* Diencephalon, *SMG* Supramarginal Gyrus, *MTG* Middle Temporal Gyrus, *ITG* Inferior Temporal Gyrus.

### Music training effects on rhythm and motor processes

**Figure 1:**
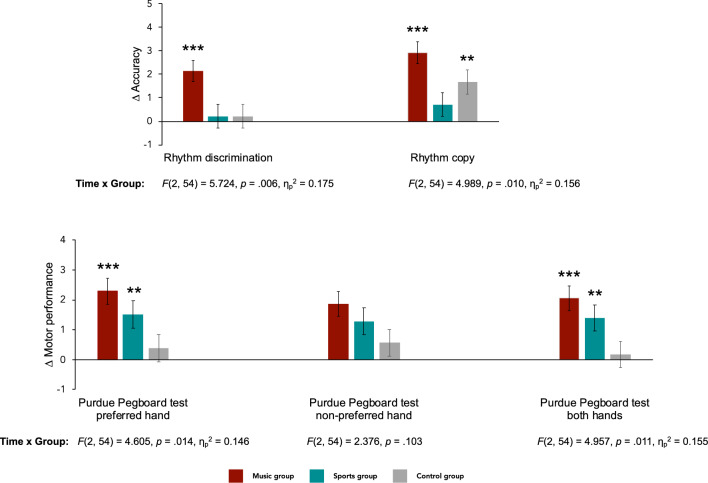
Pre- to post-test performance change (Δ) in rhythm and motor skills in the music, sports, and control groups. Time by group interactions are presented for each variable and subsequent significant pre- to post-test performance changes are indicated by asterisks (* *p* < .05; ** *p* < .01; *** *p* < .001). Error bars represent standard error.

Figure 1 shows the improvements from pre- to post-test in the rhythm and motor tasks, in each group; full descriptive statistics on the pre- and post-test scores are given in Supplementary Table S1. At pre-test, the groups did not differ in rhythm copy, *F*(2,54) = 0.073, *p* = .930, nor in motor performance: for the Purdue Pegboard test with the preferred hand, *F*(2,54) = 1.939, *p* = .154, the non-preferred hand, *F*(2,54) = 0.451, *p* = 0.640, and both hands, *F*(2,54) = 1.157, *p* = .322. They differed, though, in rhythm discrimination *F*(2,54) = 3.506, *p* = .037, η_p_^2^ = 0.115, where the control group reached better performance than the music group (*p* = .032, *d* = 0.829; no other pairwise comparisons were significant,* p* ≥ .247 ). Considering performance before and after training, we found a pattern of superiority of the music group in motor learning as in our previous results with a larger sample^40^. Time by Group interactions were significant for the Purdue Pegboard test with the preferred hand, *F*(2, 54) = 4.605, *p* = .014, η_p_^2^ = 0.146, and with both hands, *F*(2, 54) = 4.957, *p* = .011, η_p_^2^= 0.155, but not for the non-preferred hand, *F*(2, 54) = 2.376, *p* = .103. With the preferred hand, the music group showed the greatest improvement from pre- to post-test, *M* = 2.286, *SE* = 0.425, *p* < .001, *d* = 1.337 (sports group: *M* = 1.500, *SE* = 0.459, *p* = .002, *d* = 0.701; control group: *M* = 0.389, *SE* = 0.459, *p* = .401), and outperformed sports (*M* = 1.413, *SE* = 0.604, *p* = .046, *d* = 0.687) and control groups (*M* = 1.413, *SE* = 0.604, *p* = .046, *d* = 0.851) at post-test. The sports and control groups did not differ at post-test (*p* = 1.000). With both hands, the music group also presented the greatest motor enhancement from pre- to post-test, *M* = 2.048, *SE* = 0.409, *p* < .001, *d* = 1.303 (sports group: *M* = 1.389, *SE* = 0.442, *p* = .003, *d* = 0.778; control group: *M* = 0.167, *SE* = 0.442, *p* = .707). At post-test, the music group did not differ from the sports group, *M* = 0.952, *SE* = 0.562, *p* = .192, nor did the control one, *M* = 1.341, *SE* = 0.562, *p* = .062. The sports and control groups did not differ at post-test (*p* = .507). For rhythm skills, we found a similar pattern of superiority of the music group. Time by Group interactions were significant for rhythm discrimination, *F*(2, 54) = 5.724, *p* = .006, η_p_^2^ = 0.175, and rhythm copy, *F*(2, 54) = 4.989, *p* = .010, η_p_^2^ = 0.156. In rhythm discrimination, the music group improved from pre- to-post-test, *M* = 2.143, *SE* = 0.451, *p* < .001, *d* = 0.796, but no significant differences were observed in the sports, *M* = .222, *SE* = 0.487, *p* = .650, and control groups, *M* = .222, *SE* = .487, *p* = .650. At post-test, there were no between-group differences (*p*_s_ > .05). In rhythm copy, the music group had the greatest pre- to post-test improvement, *M* = 2.905, *SE* = 0.473, *p* < .001, *d* = 0.721; the control group improved less strongly, *M* = 1.667, *SE* = 0.511, *p* = .002, *d* = 0.422, and the sports group did not improve, *M* = .722, *SE* = 0.511, *p* = .163. As in discrimination, at post-test the groups did not differ from each other (*p*_s_ > .05). Additional details and analyses are given in Supplementary Information.

**Figure 2:**
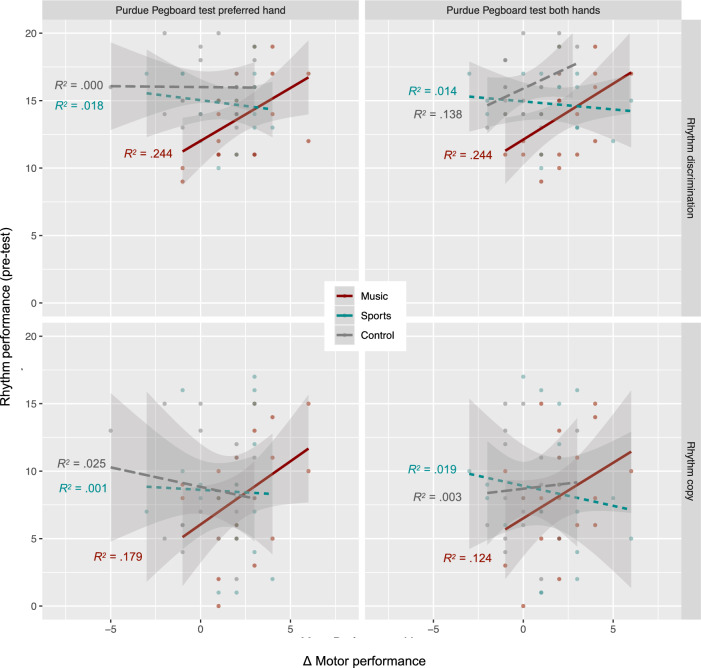
Scatterplots depicting the correlation between the pre-test scores of rhythm discrimination (upper panel) and rhythm copy (lower panel), and the performance change (Δ) in the Purdue Pegboard test with preferred hand (left panel) and both hands (right panel), in the music, sports, and control groups.

Pearson correlations were computed between the pre-test scores of rhythm skills (discrimination and copy) and the gain scores of the motor measures modulated by music training, that is, the performance on the Purdue Pegboard with the preferred hand and with both hands (Fig. 2). In the music group, better ability to discriminate rhythm at pre-test correlated with higher gains in the Purdue Pegboard with the preferred hand, *r* = .494, 95% CI [0.079, 0.763], *p* = .023, and with both hands, *r* = .494, 95% CI [0.079, 0.763], *p* = .046. Performance on rhythm copy at pre-test did not correlate with motor gains (*p*s > .05). No correlations were found between these variables in the sports and control groups (*p*s > .05). There were also no correlations between rhythm and motor skills in the whole group before training (*p*s > .53; Supplementary Table S2).

**Figure 3:**
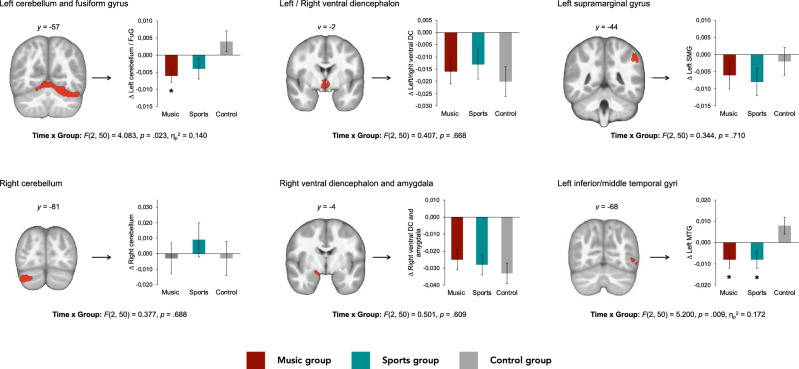
Pre- to post-test volume change (Δ) in the gray matter correlates of rhythm discrimination in the music, sports, and control groups, after controlling for TIV (pre- to post-test differential), age, sex, and handedness. Time by group interactions are presented for each cluster and subsequent significant pre- to post-test volume changes are indicated by asterisks (* *p* < .05). Error bars represent standard error.

Modulatory effects of training on the gray matter correlates of rhythm discrimination were then examined. Music, sports, and control groups did not differ in total intracranial volume (TIV), neither at pre-test nor in pre- to post-test change. They also did not differ in the gray matter volume of the rhythm discrimination correlates at pre-test (*p*s > .05; see Supplementary Table S3 for details about these analyses and for absolute gray matter volumes of these correlates at pre- and post-test). Figure 3 shows the pre- to post-test gray matter volume changes in the rhythm discrimination brain correlates in the each group. The gray matter volume of the cluster comprising the left cerebellum and the fusiform gyrus did not show significant main effects of Time, *F*(1, 50) = 3.643, *p* = .062, η_p_^2^ = 0.068, or Group, *F*(2, 50) = 0.273, *p* = .762, but the Time by Group interaction was significant, *F*(2, 50) = 4.083, *p* = .023, η_p_^2^ = 0.140. As depicted in Fig. 3, the music group had a significant decrease in the gray matter volume of this cluster from pre- to post-test, *M* = − 0.006, *SE* = 0.002, *p* = .026, *d* = 0.131. A similar trend was visible in the sports group, but it was not significant, *M* = -0.004, *SE* = 0.003, *p* = .144. The control group showed a non-significant change in the opposite direction, *M* = 0.004, *SE* = 0.003, *p* = .112. At post-test, the groups did not differ in the gray matter volume of this cluster (*p*s > .05). Gray matter volume in the cluster of the left inferior/middle temporal gyri did not differ over time or across groups (for Time, *F*(1, 50) = 0.659, *p* = .421; for Group, *F*(2, 50) = 0.025, *p* = .975), but the interaction Time x Group was significant, *F*(2, 50) = 5.200, *p* = .009, η_p_^2^ = 0.172: it decreased significantly from pre- to post-test in the music group, *M* = − 0.008, *SE* = 0.004, *p* = .041, *d* = 0.109, and in the sports group, *M* = − 0.008, *SE* = 0.004, *p* = .042, *d* = 0.125. The control group had a non-significant increase in the gray matter volume of this cluster, *M* = 0.008, *SE* = 0.004, *p* = .06. At post-test, the groups did not differ in the gray matter volume of this cluster (*p*s > .05). For the remaining clusters, no significant main effects nor interactions were observed (*p*s > .05).

**Figure 4:**
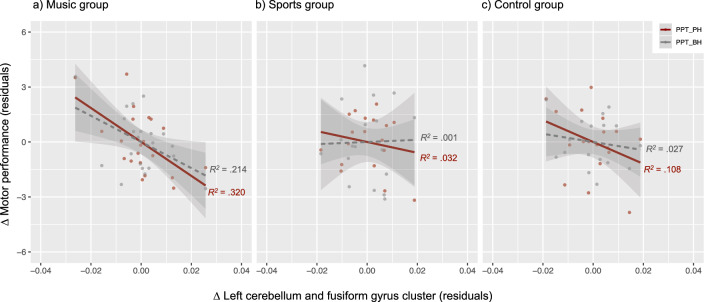
Scatterplots depicting the correlation between motor performance change (Δ) in the Purdue Pegboard test with the preferred hand (PPT-PH) and both hands (PPT-BH) and the pre- to post-test gray matter volume change (Δ) in the left cerebellum and fusiform gyrus cluster, in the music (**a**), sports (**b**), and control (**c**) groups, after removing the effects of TIV (pre- to post-test difference), age, sex, and handedness.

Figure 4 illustrates the correlations between the pre- to post-test changes in the gray matter volume of the left cerebellum and fusiform gyrus cluster with the motor improvements in the Purdue Pegboard test with the preferred hand and both hands, in the music (Fig. 4a), sports (Fig. 4b) and control groups (Fig. 4c). In the music group, the change in gray matter was negatively correlated with the motor improvement in the Purdue Pegboard test with the preferred hand, *r* =  − 0.566, 95% CI [− 0.807, − 0.028], *p* = .036; with both hands, the correlation did not reach significance, *r* =  − .462, 95% CI [− 0.838, − 0.275], *p* = .062. No correlations were found in the sports and control groups (*p*s > .05).

We repeated the group-based analyses including SES as a covariate, and the results were similar.

## Discussion

In this study, we focus on the link between rhythm, motor processes, and music. Rhythm and motor processes have been extensively examined in relation to music experience and cognition, typically regarding differences between musicians and non-musicians^24,28,31^ and training-related improvements in one or the other^36,38,40^. Less is known about the rhythm-motor interplay during music training proper, its neural underpinning, and impact on training-related effects. Addressing these questions is relevant on theoretical and practical grounds to better devise and implement training or intervention programs targeting motor learning and rehabilitation. Here, 8-year-old children participated in a training study where they completed rhythm and motor tasks, as well as structural MRI scans before and after a music or a sports program, or no specific training (passive control group). We report five main findings: (1) better ability to discriminate rhythm before training (i.e., rhythm predisposition) was associated with less gray matter volume in regions of the cerebellum, fusiform gyrus, supramarginal gyrus, ventral diencephalon, amygdala, and inferior/middle temporal gyri; (2) music training improved motor dexterity and coordination of the preferred hand and the simultaneous use of both hands, as well as rhythm perception and production skills; (3) predisposition to perceive rhythms was related to greater motor improvements in manual dexterity in the music group, but not in the sports or control groups; (4) music training modulated the gray matter correlates of rhythm perception in the left cerebellum and fusiform gyrus by inducing a significant decrease in gray matter volume that was not observed in the sports and control groups, and (5) this gray matter volume change correlated negatively with the motor improvement in the preferred hand of musically-trained children.

Children’s predisposition to perceive rhythm was associated with less gray matter volume in several brain regions, including auditory and motor areas that are part of the widespread bilateral cortico-subcortical brain network typically engaged in rhythm cognition^8^. That less, and not more, gray matter volume was related to better function is surprising, especially because it occurred in regions where gray matter volume peaks relatively late in childhood, such as the anterior and superior posterior lobes of the cerebellum (13.5–18.2 years)^82^. However, such a negative relationship between brain structure and cognition is not entirely new: it has been reported in global indices of structural brain maturation^83^ and in cortical^84,85^, subcortical^86^, and cerebellar^87,88^ brain measures. Indeed, findings from MRI studies suggest that brain maturation occurs during development through continuous myelination—a progressive growth of white matter tissue—together with a concurrent decline in gray matter volume, which may be partially associated with neuronal pruning^89^. In line with this view, an interpretation of the negative rhythm-gray matter volume relation that emerged in this study is that children with better pre-training rhythm perception had a more mature developmental trajectory of the cerebellum.

The fourth and fifth findings of this study agree with the notion that bigger is not necessarily better. Musically-trained children showed a significant decrease in the gray matter volume of a cluster including the left cerebellum and fusiform gyrus—a brain correlate of rhythm perception—and such a decrease did not happen in the sports and control groups. Importantly, this music-related volumetric change was coupled with modulations in the motor domain: the magnitude of the loss was associated with greater motor improvement in the preferred hand. In other words, the decrease in gray matter volume reflected more efficient processing. Note that the region we found here subtending rhythm predisposition and music-related motor learning has been associated with responsiveness to auditory rhythms, regardless of whether actual actions or perceptual representations triggered the response^19^. It is also revealing that the loss of cerebellar gray matter volume in musically trained children accords well with findings from recent studies showing training-related volume losses in regions grossly overlapping with the ones we observed^31,90^. Baer and colleagues^31^ found that early-trained musicians (onset of training before age 7) had smaller volumes in bilateral cerebellar white matter and right lobules IV, V, and VI than their late-trained peers, and that smaller volume was associated with better finger tapping performance. And recently Shenker et al.^90^ reported volumetric reductions in cerebellar regions IV, V, and VI, but, differently from Baer et al.^31^, the reductions in lobules IV and VI were in the vermal area and in the left hemisphere. These authors suggest that a reduction in cerebellar volume, together with an increase in the cortical volume of motor areas, is part of a widespread structural reorganization in cortico-cerebellar networks due to training; they support this interpretation with findings coming from longitudinal studies of sensorimotor learning in mice^91,92^ and evidence on the anatomical and functional link between cortical and cerebellar regions^93,94^. By unveiling a specific modulation of the left cerebellum and fusiform gyrus subtending the enhancement of motor coordination induced by music training, we add further evidence in favor of the notion of a structural reorganization in cortico-cerebellar networks driven by experience.

At the behavioral level, we found that children who had music training improved their rhythm and motor skills more than those in the sports and control groups. Considering that rhythm and motor processes are directly implicated in music performance, these findings are hardly unexpected, and indeed they match extant evidence on the benefits of music training for rhythm skills^36,37^ and motor performance^36,38^. Importantly, better ability to discriminate rhythm before training was associated with greater improvement in motor performance with the preferred hand and with both hands in the music group, but not in sports or control groups. This finding suggests that rhythm predisposition combined with music training induces fine motor learning. It concords with evidence from recent studies with Parkinson's patients demonstrating that gait rehabilitation through music depended on rhythm skills^75,78^. However, contrary to our expectations and the idea of a rather unspecific rhythm-motor link, we failed to find a correlation between rhythm and motor skills before training. Why did the link arise, in our data, only with music training? It is known that music engages auditory and motor networks^12,23^ and that musicians present stronger auditory-motor coupling than non-musicians^17,69,70^. So, it is conceivable that music training boosts cross-modal plasticity and forges (or enhances) the connection between rhythm abilities and motor learning. This might have happened with the children who participated in our study, and this is how we interpret our results. But only future studies can clarify whether the lack of a general correlation between rhythmic and motor performance was related to the tasks used here or whether it is characteristic of the rhythm-motor interplay during development.

A limitation of this study is the relatively small number of participants. Our behavioral sample was larger (*N* = 74), but only 57 children met the criteria for this study, i.e., having completed both behavioral and neuroimaging assessment protocols at pre- and post-test. The reason is that neuroimaging studies with children set non-trivial logistical challenges for recruiting and keeping participants, particularly in the context of a middle- to low-income community like the one involved in this study, where 55% of the children attending the participating schools received free or reduced-price meals, and more than 70% of parents or legal guardians had less than secondary education (only 7% had higher education). Considering these aspects and that similar previous studies had similar sample sizes^36,51^, we believe that the results reported here provide a useful contribution to our knowledge on the effects of music training on behavior and brain plasticity, and on how individual predispositions can constrain experience-related enhancements.

In sum, the present longitudinal training study with children revealed an interaction between rhythm predisposition and experience/music training-related motor learning, supported by behavioral and brain evidence. The study was conducted in an ecologically valid environment comparing the effects of music training with similarly challenging training in sports and a no-specific control condition. We found that music training improved motor performance (and also rhythm), and that the magnitude of the improvement depended on the ability to perceive rhythm before training (i.e., better rhythm predisposition, more significant improvements). Thus, music programs are likely to be more effective in individuals with rhythm perception predisposition. Notably, a region comprising the left cerebellum and fusiform gyrus underpinned the link between rhythm predisposition and motor improvements induced by music training. To the best of our knowledge, this is the first study demonstrating with behavioral and brain evidence that music-related motor learning results from the interaction between predisposition and experience. These findings have implications for models of music-related plasticity and rhythm cognition and also for debates on how learning is modulated by predispositions/interindividual differences vs. experience.

## Methods

### Ethics statement

The study was approved by the ethics committee of the Faculty of Psychology and Education Sciences at the University of Porto (FPCEUP 2015.1.23) and by the managing boards of the schools where the children were recruited from. The work was conducted in accordance with the Declaration of Helsinki. Written informed consent was obtained from the children’s parents or legal guardians, who also completed an MRI safety form to ensure a safe scanning of the participants. Children gave their verbal assent before data collection started.

### Participants

Fifty-seven Portuguese children participated in the study (33 girls, *M* age = 8.285 years, range = 7.750–9.250, *SD* = 0.325). All were third graders from five elementary public schools in a middle- to low-income community in Northern Portugal. This sample was drawn from a larger group of children (*N* = 74) who were enrolled in a project looking at music training, reading, auditory processing, and brain plasticity^40,95,96^, and only included children who had completed behavioral and MRI assessments at pre- and post-test (*N* = 60); three participants had to be excluded due to poor quality of the neuroimaging data. At the beginning of the study, children were pseudorandomly allocated to music or sports (basketball) training, or no training (control group). Parents reported that children had no prior experience in instrumental music practice or basketball (see Design and Procedure). The groups did not differ in age, *F*(2, 54) = 1.433, *p* = .247, sex, *χ*^2^(2) = 1.554, *p* = .460, handedness, *F*(2,54) = 0.463, *p* = .632, full-scale IQ, *F*(2,54) = 0.357, *p* = .702, and attendance of extracurricular activities, *χ*^2^(2) = 3.928, *p* = .140. Regarding SES, the control group had fewer children from lower SES (n = 3) than the music (n = 11) and sports (n = 10) groups, χ^2^(2) = 7.024, *p* = .030. Please see Table 2 for detailed information.

**Table 2:** Demographic and cognitive characteristics of children in the music, sports and control groups before training.

	Music group (n = 21)	Sports group (n = 18)	Control group (n = 18)
Sex (girls/boys)	10/11	11/7	12/6
SES (lower/higher)	11/10	10/8	3/15
Extracurricular activities (yes/no)	10/11	12/6	14/4
Age (years)	8.317 ± 0.315	8.181 ± 0.356	8.352 ± 0.296
Handedness^a^	89.048 ± 29.395	81.944 ± 34.689	90.000 ± 14.653
Full-scale IQ^b^	95.714 ± 12.471	93.500 ± 13.080	97.333 ± 15.500

*SES* Socioeconomic status.

^a^Edinburgh Handedness Inventory; ^b^Wechsler Intelligence Scale for Children—WISC III, Portuguese version.

### Behavioral assessment

The behavioral assessment protocol included measures of handedness, general intellectual ability, and rhythm and fine motor abilities. Handedness was assessed with Cohen’s^97^ revised version of the Edinburgh Handedness Inventory^98^, and general intellectual ability with the Wechsler Intelligence Scale for Children—3rd Edition (WISC—III)^99^. Rhythm abilities were tested with Moore’s^100^ revised version of the rhythm discrimination and rhythm copy subtests of the Musical Aptitude Tests (MATs)^101^. Fine motor abilities were assessed with the Purdue Pegboard test^102^.

The rhythm discrimination test from MATs is a forced-choice same-different judgment task: the child is presented with two rhythmic sequences and has to decide whether they sound exactly the same or not. It consists of 2 practice and 20 test trials, half the same and half different, and the score is the number of correct responses (20 maximum). The rhythm copy task is a measure of rhythm production. It consists of 3 practice trials followed by 20 test trials. In each trial, the child hears a rhythmic sequence that she must reproduce using a prespecified key on a MIDI keyboard; the reproduced sequence is recorded in GarageBand and assessed offline (see below Design and Procedure). Each rhythm is played only once, and the score is the number of correct responses (20 maximum). Scoring is carried out independently by two music experts who assess whether the child has reproduced the rhythmic sequence correctly; in case of dissension, they discuss until a final agreement is reached. In both tests, the difficulty of the trials increases, progressing from short and simple sequences to longer and more complex ones. MATs also include measures of melodic discrimination and rapid temporal processing skills (i.e., note number detection task); the data and longitudinal results for these variables are available at https://osf.io/y3p97/?view_only=4a5da2f905c64616aa6660fb03fabbd4.

The Purdue Pegboard test is a well-established measure of manual dexterity and bimanual coordination. It is performed on a board with two vertically aligned series of 25 holes, and the task is to insert in the holes as many pegs as possible within 30 s. The test includes two unimanual subtests, with the preferred hand and the non-preferred hand, and one bimanual subtest performed with both hands simultaneously. In the unimanual subtests, the score is the number of correctly inserted pegs, and in the bimanual subtest it is the number of correctly inserted peg pairs.

### Brain imaging

#### MRI acquisition

Anatomical data were acquired in a 1.5T Siemens Magnetom Sonata Maestro Class (Siemens Medical Systems, Erlangen, Germany). We used a T1-weighted sequence (3D magnetization prepared rapid gradient echo, MPRAGE) with the following parameters: 1680 ms repetition time, 4.12 ms echo time, 8° flip angle; 160 contiguous sagittal slices, 250 × 250 mm^2^ field-of-view. A 1mm isotropic voxel was used to accomplish a good differentiation between tissue types (gray- and white-matter, cerebrospinal fluid). Head motion during scanning was reduced with cushions around the head and a strap on the forehead.

#### Image processing

T1-weighted images were preprocessed through the SPM12 package (http://www.fil.ion.ucl.ac.uk/spm) and the CAT12.6 r1450 toolbox^103^, running under MATLAB R2015a (Mathworks, Sherborn, MA). The raw data were manually inspected for individual and scanner-based artifacts (e.g., motion), and the origin was manually set on the anterior commissure according to the Montreal Neurological Institute (MNI) spatial coordinate system. To preprocess the images, tissue probability maps were generated using the Template-O-Matic toolbox (http://dbm.neuro.uni-jena.de/software/tom/) with age and sex as defining variables^104^. The age at the midpoint between pre- and post-test was used as reference (*M* = 8.575 years, *SD* = 0.325). A study-specific template was also created using the Diffeomorphic Anatomical Registration Through Exponentiated Lie Algebra, DARTEL^105^. Then, the images were inspected for poor quality and incorrect preprocessing using the check sample homogeneity function of CAT12. As mentioned, three subjects were excluded due to poor data quality (i.e., cumulative low within- and between-subject correlations). Finally, the modulated gray matter volumes were smoothed with a Gaussian kernel of 8 mm full width at half maximum. TIV (the sum of gray matter, white matter, and cerebrospinal fluid volumes) was extracted using the estimation module in CAT12.

### Design and procedure

The study included a pre-test, 6-month training, and a post-test. At the pre-test, children completed the behavioral assessment protocol (handedness, general intellectual ability, rhythm, and fine motor abilities) and an MRI session in which the structural MRI was acquired. At post-test, rhythm and motor abilities were again tested, and the neuroimaging data was once more collected. The behavioral assessments were conducted in individual sessions in a quiet room of the children’s school; the WISC-III battery was administered in a single session by an experienced child psychologist, and rhythm and motor abilities were assessed by a trained research assistant in later sessions. In the rhythm tasks, stimuli were delivered via headphones (Sennheiser HD 201) connected to an Apple MacBook Pro laptop. The children’s responses in the rhythm discrimination task were registered on each participant’s response sheet, and in the rhythm copy task they were recorded in a GarageBand audio file (https://www.apple.com/uk/mac/garageband/). In the Purdue Pegboard test, the hand order (preferred vs. non-preferred) was counterbalanced within each group; the bimanual subtest was always conducted after the unimanual subtests. A training trial was performed before each subtest, and the time was counted from the moment the child picked up the first peg. MRI scans were acquired in a 20-min session at the neuroimaging center.

Music and sports training programs started right after the pre-test assessment and went on for approximately 24 weeks, from October to May, with interruptions for school holidays, in 90-min sessions twice a week. Children in the passive control group were engaged in different extracurricular activities but not in systematic music or basketball training. Both types of training were made available in the following academic year so that interested children from the passive control group would be able to participate in them if desired.

Before the collection, parents completed a sociodemographic questionnaire including questions on the children’s prior experience with music, sports, or other activities with which they might have been engaged. Information about socioeconomic support was gathered from school records, i.e., whether children were provided with free or reduced-price meals or had no such support. This data was used as a proxy for lower and higher SES, respectively.

### Training

The music and sports training programs took place in collective sessions (90’ twice a week, as mentioned before). They included activities adapted to third-grade children with no prior formal instruction on music or basketball and were analogous in difficulty, expected progression, and motivational aspects. The music training program used an Orff-based approach and was structured into four domains: music awareness, elementary music concepts, rhythm and pitch skills, and instrumental and vocal performance. The sports training program consisted of basketball practice. It was also structured into four domains: physical fitness, game-relevant motor coordination (upper and lower limbs, eye-hand coordination), team sports concepts and schemes, and tactical planning. The activities conducted in the music and sports training typically embedded at least two of the training domains, and included a declarative (musical concept) and a practical component. For a detailed description of the music and basketball training programs, please see Martins et al.^40^

### Data analysis

*First,* we performed regression analyses using the multiple regression model implemented in SPM12 to inspect for brain regions associated with rhythm skills (discrimination and copy) in the whole sample (*N* = 57). Age, sex, and handedness were added as covariates of no interest in the models. A global scaling adjustment for TIV (*M* = 1420.386 cm^3^, *SD* = 144.431) was employed, and an absolute threshold masking excluded voxels with intensities below 10%. We used a gray matter mask created on the basis of the Shooting template of CAT12 toolbox to ensure that only gray matter volume was inspected in the analysis. A combined analysis of the height and size of the effects was accomplished using a threshold-free cluster enhancement (TFCE)^106^ implemented in the TFCE toolbox (http://dbm.neuro.uni-jena.de/tfce/). Statistical inference was established via False Discovery Rate correction (FDR, *p* < 0.05; *k* > 20) for multiple comparisons using nonparametric permutation testing (10,000 permutations). Permutation testing was calculated using the Freedman-Lane method^107^. A template created based on our own sample (modulated and warped images) was used for the visualization of the results. *Second*, we performed one-way ANOVAs to check for group differences in rhythm and motor skills before training. Training effects were then examined by calculating repeated measures ANOVAs with Group (music, sports, and control) as between-subjects factor and Time (pre- and post-test) as within-subjects factor. Differences between groups at each time point and progression across time points were tested using post hoc pairwise comparisons. For each pairwise comparison, we report the mean difference *M*, standard error *SE*, and *p*-value; when the difference between groups or/and time points was significant, we also report the magnitude of the effect using Cohen’s *d*. *Third*, we computed Pearson correlations between pre-test rhythm skills and pre- to post-test motor changes and examine whether the benefits of music training on motor performance were related to the ability to process rhythm before training. We also calculated analogous correlation coefficients for the sports and control groups and for the association between rhythm and motor skills prior to training in the whole group (*N* = 57). *Fourth*, we examined potential modulatory effects of training on data-driven regions of interest (ROI). The ROIs were derived from the initial multiple regression analysis on the brain correlates of rhythm skills. The REX toolbox for SPM (http://web.mit.edu/swg/software.htm) was used to extract individual pre- and post-test gray matter volumes at these ROIs. One-way ANOVAs were performed to inspect for group differences in TIV (pre-test and pre- to post-test differential) and in the gray matter volume of the defined ROIs. The effects of training were then analyzed through repeated measures ANOVAs with Group (music, sports, and control) as between-subjects factor and Time (pre- and post-test) as within-subjects factor. TIV (pre- to post-test differential), age, sex, and handedness were added as covariates of no interest in all models. Differences between groups at both time points and progression across time points were tested using post hoc pairwise comparisons. For each comparison, we report the mean difference *M*, standard error *SE*, and *p*-value; as before, we also report Cohen’s *d* for significant differences. *Lastly*, we used Pearson correlations to examine the relationship between gray matter volume changes in regions showing modulatory effects of music training and changes in motor performance showing an advantage of music training (when compared to sports and control groups). TIV (pre- to post-test differential), age, sex, and handedness were added as covariates of no interest. As the groups differed on SES, the analyses in which the groups were compared were repeated covarying for this variable. Holm-Bonferroni corrected *p*-values are presented for all the multiple comparisons conducted.

The full dataset can be found here: https://osf.io/y3p97/?view_only=4a5da2f905c64616aa6660fb03fabbd4.

## Supplementary Information

Supplementary Information.

## Data Availability

We would like to make available all the data discussed in this work; however, due to the sensitivity of medical data (MRI data), the raw data sets are available from the corresponding author upon reasonable request, depending on agreements not to share these data publicly. The data that can be made available is accessible at https://osf.io/y3p97/?view_only=4a5da2f905c64616aa6660fb03fabbd4 (after an anonymization process).
