# Supplementary material for: Individual differences in rhythm perception modulate music-related motor learning: a neurobehavioral training study with children
Source: Sci Rep. 2023 Dec 6;13:21552. doi: 10.1038/s41598-023-48132-2 (PMC10700636; doi:10.1038/s41598-023-48132-2)
Supplement: Supplementary file 1 — Supplementary Information. [file 41598_2023_48132_MOESM1_ESM.docx]

**Individual differences in rhythm perception modulate music-related motor learning: A neurobehavioral training study with children**

Marta Martins, Ana Mafalda Reis, Christian Gaser, and São Luís Castro

**Supplementary information**

Performance on the **Purdue Pegboard Test, preferred hand**, improved from pre- to post-test, *M* = 1.392, *SE* = 0.259, *p* < .001, *d* = 0.766, main effect of Time *F*(1, 54) = 28.912, *p* < .001, η_p_^2^ = 0.349. No main effect of Group was reached, *F*(2, 54) = 2.231, *p* = .117. Importantly, the interaction of Time with Group was significant, *F*(2, 54) = 4.605, *p* = .014, η_p_^2^ = 0.146. The greatest increase from pre- to post-test occurred in the music group, *M* = 2.286, *SE* = 0.425, *p* < .001, *d* = 1.337; the sports group also showed a significant improvement, though not so marked, *M* = 1.500, *SE* = 0.459, *p* = .002, *d* = 0.701, but in the control group the increase was not significant, *M* = 0.389, *SE* = 0.459, *p* = .401. Looking at the differences between groups at post-test, the superiority of the music group emerged again: it had a significant advantage when compared to the sports, *M* = 1.413, *SE* = 0.604, *p* = .046, *d* = 0.687, and the control group, *M* = 1.413, *SE* = 0.604, *p* = .046, *d* = 0.851, that did not differ from each other (*p* = 1.000). With the **non-preferred hand**, the performance on the Purdue Pegboard Test also improved significantly from pre- to post-test, *M* = 1.230, *SE* = 0.247, *p* < .001, *d* = 0.676, main effect of Time, *F*(1, 54) = 24.827, *p* < .001, η_p_^2^ = 0.315. No main effect of Group, *F*(2, 54) = 1.005, *p* = .373, nor the interaction Time x Group, *F*(2, 54) = 2.376, *p* = .103, reached significance. **Bimanual performance** improved significantly from pre- to post-test, *M* = 1.201, *SE* = 0.249, *p* < .001, *d* = 0.706, main effect of Time, *F*(1, 54) = 23.279, *p* < .001, η_p_^2^ = 0.301. Time interacted with Group, *F*(2, 54) = 4.957, *p* = .011, η_p_^2^ = 0.155: the music and sports groups improved from pre- to post-test, but the increase was greater in the music, *M* = 2.048, *SE* = 0.409, *p* < .001, *d* = 1.303, than in the sports group, *M* = 1.389, *SE* = 0.442, *p* = .003, *d* = 0.778; the control group did not improve, *M* = 0.167, *SE* = 0.442, *p* = .707. The main effect of Group was not significant, *F*(2, 54) = 0.958, *p* = .390. At post-test, the music group showed a significant advantage compared to the control group, but this advantage did not survive the Holm-Bonferroni correction for multiple comparisons, *M* = 1.341, *SE* = 0.562, *p* = .062; the difference to sports group did not reach significance, *M* = 0.952, *SE* = 0.562, *p* = .192. No significant differences were found between sports and control groups at post-test, *M* = 0.389, *SE* = 0.583, *p* = .507.

Regarding the effects of training on rhythm skills, the performance on **rhythm discrimination** improved from pre- to post-test, *M* = 0.862, *SE* = 0.275, *p* = .003, *d* = 0.365, main effect of Time, *F*(1, 54) = 9.867, *p* = .003, η_p_^2^ = 0.154. The main effect of Group was not significant, *F*(2, 54) = 1.989, *p* = .147, but the interaction Time x Group was, *F*(2, 54) = 5.724, *p* = .006, η_p_^2^ = 0.175. The greatest increase from pre- to post-test occurred in the music group, *M* = 2.143, *SE* = 0.451, *p* < .001, *d* = 0.796, and no significant differences were observed in the other groups (sports group: *M* = .222, *SE* = 0.487, *p* = .650; control group: *M* = .222, *SE* = .487, *p* = .650). The groups did not differ at post-test (*p*s > .05). Because the rhythm discrimination pre-test scores differed significantly between the music and control groups, we performed an ANCOVA to check whether the post-test means, adjusted for pre-test scores, differed between groups. This established a significant effect of group in the rhythm discrimination post-test scores after controlling for the pre-test scores, *F*(2, 53) = 3.569, *p* = .035, η_p_^2^ = 0.119: the music group had better rhythm discrimination than the sports group, *M* = 1.476, *SE* = 0.557, *p* = .032, *d* = 0.851, but not than the control group, *M* = 0.915, *SE* = 0.584, *p* = .247. Performance on the **rhythm copy** task improved from pre- to post-test, *M* = 1.765, *SE* = 0.288, *p* < .001, *d* = 0.422, main effect of Time, *F*(1, 54) = 37.619, *p* < .001, η_p_^2^ = 0.411. Time interacted with Group, *F*(2, 54) = 4.989, *p* = .010, η_p_^2^ = 0.156. The music group showed a significant improvement from pre- to post-test, *M* = 2.905, *SE* = 0.473, *p* < .001, *d* = 0.721, and the control group also, although less markedly, *M* = 1.667, *SE* = 0.511, *p* = .002, *d* = 0.422. The sports group did not improve, *M* = .722, *SE* = 0.511, *p* = .163. The main effect of Group was not significant, *F*(2, 54) = 0.209, *p* = .812. No differences were found between the groups at post-test (*p*s > .05).

Analogous analyses were computed adding SES as a covariate and the results were similar.

**Supplementary Table S1.** Pre- and Post-test Rhythm and Motor Scores for the Music, Sports, and Control Groups (*N* = 57). Minimum and maximum values in parentheses.

| Variable | Music group (*n* = 21) | Sports group (*n* = 18) | Control group (*n* = 18) |
| --- | --- | --- | --- |
| Pre-test assessment |  |  |  |
| Rhythm discrimination | 13.810 ± 2.977 (9 - 19) | 14.778 ± 2.365 (10 - 19) | 16.000 ± 2.249 (13 - 20) |
| Rhythm copy | 8.190 ± 4.155 (0 - 15) | 8.500 ± 5.090 (1 - 17) | 8.722 ± 3.862 (2 - 15) |
| PPT - preferred hand | 12.238 ± 1.609 (9 - 15) | 11.611 ± 2.004 (8 - 15) | 12.722 ± 1.447 (11 - 16) |
| PPT - non-preferred hand | 11.476 ± 1.167 (9 - 13) | 11.111 ± 1.451 (9 - 14) | 11.611 ± 2.200 (8 - 16) |
| PPT - both hands | 8.905 ± 1.670 (6 - 12) | 8.611 ± 1.685 (5 - 12) | 9.444 ± 1.653 (7 - 14) |
| Post-test assessment |  |  |  |
| Rhythm discrimination | 15.952 ± 2.355 (12 - 20) | 15.000 ± 2.223 (11 - 18) | 16.222 ± 1.957 (12 - 19) |
| Rhythm copy | 11.095 ± 3.910 (3 - 17) | 9.222 ± 4.023 (2 - 15) | 10.389 ± 4.046 (4 - 17) |
| PPT - preferred hand | 14.524 ± 1.806 (12 - 18) | 13.111 ± 2.272 (9 - 17) | 13.111 ± 1.491 (10 - 15) |
| PPT - non-preferred hand | 13.333 ± 2.008 (10 - 18) | 12.389 ± 1.883 (9 - 16) | 12.167 ± 1.886 (9 - 17) |
| PPT - both hands | 10.952 ± 1.465 (8 - 13) | 10.000 ± 1.879 (6 - 13) | 9.611 ± 1.914 (6 - 14) |
| *Note*. PPT - Purdue Pegboard Test. | | | |

**Supplementary Table S2.** Pearson Correlations Between Motor (Purdue Pegboard Test) and Rhythm Skills Before Training (*N* = 57).

|  | Rhythm | |
| --- | --- | --- |
|  | Discrimination | Copy |
|  | *r, p* | *r, p* |
| Purdue Pegboard Test |  |  |
| Preferred hand | .00, .98 | .14, 1.00 |
| Non-preferred hand | .08, 1.00 | .23, .54 |
| Both hands | .05, 1.00 | .14, 1.00 |
| *Note.* Holm-Bonferroni corrected *p*-values. | | |

**Supplementary Table S3.** Total Intracranial Volume and Gray Matter Volume in Clusters 1 - 6 in the Music, Sports and Control Groups.

|  | Music group  (*n* = 21) | Sports group  (*n* = 18) | Control group  (*n* = 18) | *F^a^* | *F^b^* |
| --- | --- | --- | --- | --- | --- |
| Pre-test assessment | | | | |  |
| TIV | 1425.207 ± 163.617 | 1417.945 ± 128.006 | 1417.203 ± 144.188 | 0.018 | 0.132 |
| Cluster 1 | 0.595 ± 0.056 | 0.592 ± 0.056 | 0.566 ± 0.044 | 1.701 | 2.413 |
| Cluster 2 | 0.401 ± 0.048 | 0.392 ± 0.033 | 0.386 ± 0.039 | 0.730 | 0.577 |
| Cluster 3 | 0.427 ± 0.054 | 0.397 ± 0.072 | 0.381 ± 0.067 | 2.586 | 2.511 |
| Cluster 4 | 0.515 ± 0.061 | 0.513 ± 0.051 | 0.501 ± 0.069 | 0.271 | 0.081 |
| Cluster 5 | 0.437 ± 0.044 | 0.443 ± 0.039 | 0.442 ± 0.040 | 0.099 | 0.127 |
| Cluster 6 | 0.493 ± 0.094 | 0.485 ± 0.064 | 0.475 ± 0.057 | 0.289 | 0.238 |
| Post-test assessment | | | | | |
| Cluster 1 | 0.589 ± 0.056 | 0.589 ± 0.058 | 0.569 ± 0.040 | -^c^ | -^c^ |
| Cluster 2 | 0.384 ± 0.039 | 0.379 ± 0.028 | 0.367 ± 0.036 | -^c^ | -^c^ |
| Cluster 3 | 0.424 ± 0.055 | 0.390 ± 0.070 | 0.376 ± 0.061 | -^c^ | -^c^ |
| Cluster 4 | 0.511 ± 0.072 | 0.522 ± 0.059 | 0.498 ± 0.075 | -^c^ | -^c^ |
| Cluster 5 | 0.415 ± 0.043 | 0.417 ± 0.040 | 0.404 ± 0.043 | -^c^ | -^c^ |
| Cluster 6 | 0.487 ± 0.097 | 0.417 ± 0.069 | 0.481 ± 0.056 | -^c^ | -^c^ |
| ∆ TIV | 37.951 ± 47.012 | 45.864 ± 40.232 | 54.084 ± 35.344 | 0.734 | 0.716 |
| *Note.* TIV - Total Intracranial Volume; ∆ - Pre- to post-test change; *^a^*ANOVAs without co-variables; *^b^* Controlling for age, sex, and handedness in the ANOVAs testing for differences in TIV on pre-test assessment, and controlling for TIV, age, sex, and handedness on the remaining ANOVAs; *^c^* The group comparisons related to gray matter volume at post-test are discussed in the main text. | | | | | |
